# Supplementary figures and images for: Effects of Luteolin on Biofilm of Trueperella pyogenes and Its Therapeutic Effect on Rat Endometritis
Source: Int J Mol Sci. 2022 Nov 21;23(22):14451. doi: 10.3390/ijms232214451 (PMC9692790; doi:10.3390/ijms232214451)

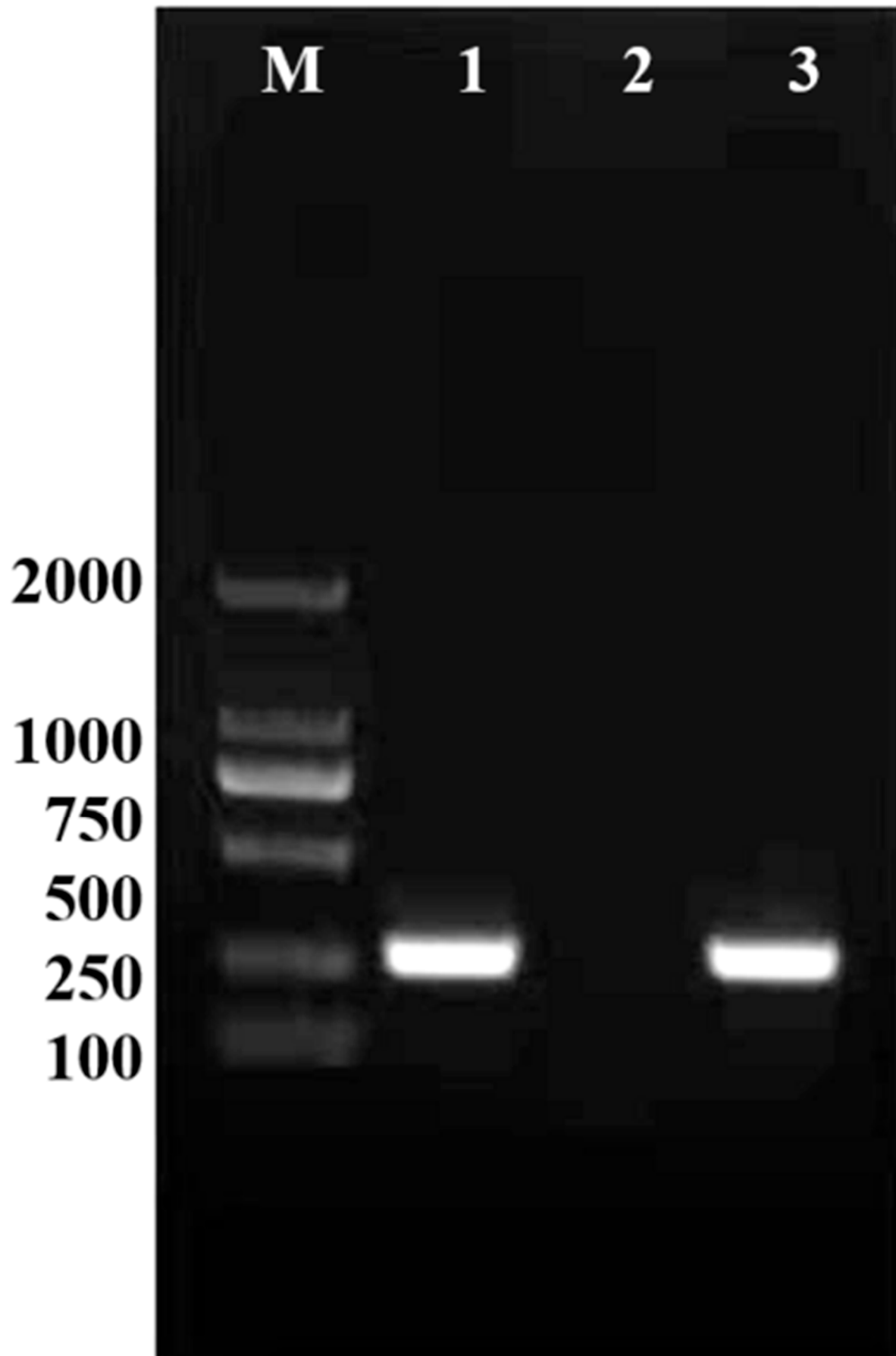

Figure S1: Screening of *plo* gene in rat intrauterine fluid of to identify *T. pyogenes* strains.

Supplement: Supplementary file 1 [file ijms-23-14451-s001.zip › ijms-1980617-supplementary.pdf]
